# Draft Genome Sequence of d-Branched-Chain Amino Acid Producer *Lactobacillus otakiensis* JCM 15040^T^, Isolated from a Traditional Japanese Pickle

**DOI:** 10.1128/genomeA.00546-13

**Published:** 2013-08-08

**Authors:** Katsumi Doi, Kazuki Mori, Yuta Mutaguchi, Kosuke Tashiro, Yasuhiro Fujino, Taketo Ohmori, Satoru Kuhara, Toshihisa Ohshima

**Affiliations:** Microbial Genetic Division, Institute of Genetic Resources, Faculty of Agriculture, Kyushu University, Fukuoka, Japana; Department of Bioscience and Biotechnology, Faculty of Agriculture, Kyushu University, Fukuoka, Japanb; Division for Arts and Science, Faculty of Arts and Science, Kyushu University, Fukuoka, Japanc

## Abstract

*Lactobacillus otakiensis* strain JCM 15040^T^ was isolated from an unsalted pickling solution used in the production of sunki, a traditional Japanese pickle. Here, we prepared a draft genome sequence for this strain consisting of 40 contigs containing a total of 2,347,132 bp, 2,310 predicted coding sequences, and a G+C content of 42.4%.

## GENOME ANNOUNCEMENT

The genus *Lactobacillus* consists of Gram-positive lactic acid bacteria (LAB). These non-spore-forming rods are catalase negative, nonmotile, and oxidase negative (1). In recent years, LAB have also attracted attention related to their ability to produce d-amino acids (2, 3). Among the LABs we examined, *Lactobacillus otakiensis* produces d-branched-chain amino acids, such as d-leucine (d-Leu), d-*allo*-isoleucine (d-*allo*-Ile), and d-valine (d-Val) (Y. Mutaguchi, T. Ohmori, H. Akano, K. Doi, and T. Oshima, submitted for publication). The N-terminal amino acid sequence of the enzyme that apparently is responsible for the production of d-branched-chain amino acids in *L. otakiensis* is completely identical to those deduced from the 4-aminobutyrate aminotransferase genes of *Lactobacillus buchneri* (Lbuc_2316 and LBUCD034_2418 of *L. buchneri* strain NRRL B-30929 and *L. buchneri* strain CD034, respectively) (Y. Mutaguchi, T. Ohmori, T. Wakamatsu, K. Doi, and T. Ohshima, submitted for publication).

*L. otakiensis* JCM 15040^T^ was isolated from an unsalted pickling solution used in the production of sunki, a traditional Japanese pickle (4). Although *L. otakiensis* is closely related to *Lactobacillus kisonensis* and *Lactobacillus rapi*, which belong to the *L. buchneri* group, *L. kisonensis* and *L. rapi* do not produce d-amino acids. It has been suggested that the 4-aminobutyrate aminotransferase genes Lbuc_2316 and LBUCD034_2418 function in the synthesis of d-branched-chain amino acids in *L. buchneri* NRRL B-30929 and *L. buchneri* CD034, respectively. Comparative genomics of strains from the *L. buchneri* group might shed light on the metabolism of d-branched-chain amino acids and facilitate in their practical application.

The sample was prepared for sequencing by growing *L. otakiensis* JCM 15040^T^ anaerobically overnight at 30°C in MRS broth (Oxoid). The genomic DNA was then extracted and purified as we described previously (5). The quantity and quality of the DNA were assessed using e-Spect, after which the prepared genome was sequenced using a 454 GS FLX system (Roche) and assembled using Newbler assembler version 2.7 software.

The genomic DNA, which includes a total of 2,347,132 bp, was sequenced using a whole-genome shotgun strategy that generated 467,404 reads and achieved approximately 62-fold coverage. Assembly of all the reads resulted in 40 contigs (>100 bp), with an N_50_ contig size of 244,550 bp. Genome annotation of the obtained scaffolds was performed using Glimmer 3.02 software and BLAST searches against a nonredundant protein sequence database. The genome of strain JCM 15040^T^ has a G+C content of 42.4%, and annotation using the GTPS (6), RDP, and Silva databases with tRNAscan-SE v.1.21 (7) and additional manual inspection revealed 2,310 predicted coding regions, 56 tRNA genes, and 5 rRNA genes.

By comparing the genome sequences of *L. otakiensis* and *L. buchneri* NRRL B-30929 using the bidirectional best hit (BBH) method with BLAST, we found that there are 1,801 orthologs with 83.82% identity. A gene encoding 4-aminobutyrate aminotransferase was located on contig 00006 as LOT_1784, which shows 97.5% identity with Lbuc_2316 of *L. buchneri* NRRL B-30929 and LBUCD034_2418 of *L. buchneri* CD034. Three other genes, LOT_0196, LOT_0493, and LOT_2079, encode racemases that function in the synthesis of d-amino acids, with d-Asp, d-Glu, and d-Ala being annotated.

### Nucleotide sequence accession numbers.

The *L. otakiensis* JCM 15040^T^ genome sequence and annotation data have been deposited in DDBJ/EMBL/GenBank under accession no. BASH01000001 to BASH01000040.
